# The Detection of *Salmonella* Enteritidis on German Layer Farms after Cleaning and Disinfection

**DOI:** 10.3390/ani13162588

**Published:** 2023-08-11

**Authors:** Pia Münster, Lars Pöppel, Ali Antakli, Doris Müller-Doblies, Dmytro Radko, Nicole Kemper

**Affiliations:** 1 Elanco Deutschland GmbH, Rathausplatz 12, 61348 Bad Homburg, Germany; pia.muenster@elancoah.com (P.M.); ali.antakli@elancoah.com (A.A.); dmytro.radko@elancoah.com (D.R.); 2 Institute for Animal Hygiene, Animal Welfare and Farm Animal Behaviors, University of Veterinary Medicine Hannover, Foundation, 30559 Hannover, Germany; lpoeppel91@googlemail.com; 3 Praxis Pöppel GmbH, Drubbelstraße 2, 33129 Delbrück, Germany; 4 Elanco Austria GmbH, Quartier Belvedere Central, Gertrude Froehlich Sandner Str. 3, 1100 Vienna, Austria; doris.mueller_doblies@elancoah.com

**Keywords:** laying hens, Germany, *Salmonella* Enteritidis, risk factor, real-time PCR

## Abstract

**Simple Summary:**

*Salmonella* is a common bacterium that can cause foodborne illness in humans. Testing poultry for *Salmonella* helps us identify contaminated products and prevents them from reaching consumers, reducing the risk of outbreaks and protecting public health. This study aimed to assess the occurrence of *Salmonella* carry-over between successive flocks of laying hens using established testing techniques. A *Salmonella* prevalence rate of 25% in the samples after cleaning and disinfection (C&D) indicates areas for improvement in C&D procedures during the service period. The study found that infectious *Salmonella* (specifically *Salmonella* Enteritidis) can persist for a long time in floor-reared production systems, which are easier to clean than caged houses. It is crucial to implement thorough C&D procedures between cycles, assess cleanliness after C&D through targeted sampling before introducing new flocks, and consider the surroundings of poultry houses when implementing hygiene measures. By incorporating *Salmonella* testing into C&D practices, poultry producers can enhance overall hygiene protocols, prevent cross-contamination, reduce the risk of contamination, and maintain a safe environment for workers and consumers.

**Abstract:**

The presence of *Salmonella* Enteritidis in poultry houses after cleaning and disinfection can pose a potential risk to public health, as *Salmonella* remains one of the most important causes of foodborne diseases. This study focused on ten German layer farms (including floor-reared and free-range systems) with a recent history of *Salmonella* Enteritidis, and samples were collected from July 2018 to March 2021 after the cleaning and disinfection process. A total of 244 swab samples were tested for the presence of *Salmonella* using real-time PCR, followed by a culture of positive samples. Results revealed that 61 out of the 244 swab samples tested positive for *Salmonella*, indicating a prevalence of 25% in the samples examined. Among the *Salmonella*-positive swab samples identified with the PCR assay, 65.6% (40 out of 61) were confirmed by the culture. Of the 40 isolates obtained from the culture, 36 were identified as *Salmonella* Enteritidis, while 4 were categorized as rough *Salmonella* strains. This study emphasizes the importance of both the surrounding area of the poultry houses in terms of infection carry-over and the meticulous implementation of cleaning and disinfection procedures to eliminate any remaining infection within the houses. To mitigate the risk of further *Salmonella* spread on layer farms, additional investigations are recommended to focus on the existing transmission pathways of *Salmonella* and their genetic diversity.

## 1. Introduction

Salmonellosis is a prominent foodborne pathogen globally, with 87,923 culture-confirmed human cases reported in the European Union in 2019 [1] and 46,623 confirmed infections documented in the United States in 2016 [2]. The World Health Organization (WHO) has estimated that *Salmonella* contributes to approximately 59,000 deaths annually worldwide [3].

Among the various serovars of *Salmonella, S.* Enteritidis (SE) has consistently been the most prevalent in many regions for several decades, primarily associated with poultry and poultry products, with eggs being the most common vehicle [4,5,6]. Starting in the early 1980s, SE, particularly phage type 4, began to emerge in European breeding and laying hen flocks, rapidly becoming the dominant serovar in poultry and causing human infections. This led to an epidemic that persisted well into the 2000s, with a similar situation occurring in various parts of the United States, albeit with different phage types [7,8,9]. Introducing measures to control the disease in breeding and laying hens, such as vaccines, improved biosecurity measures, and more effective rodent control resulted in a significant decline in human cases across Europe. These measures were initially implemented in some European Union member states in the 1990s [10]. However, despite the introduction of a stringent *Salmonella* control program for breeding chickens in 2007 and for laying hens in 2008 across Europe, human cases of salmonellosis have not continued to decrease since 2013 [11,12]. SE remains the most frequently isolated serovar from human cases in Europe, with 39,865 confirmed cases, representing 50.3% of all *Salmonella* isolates [1]. In 2019, SE was detected in 312 laying hen flocks, accounting for 24.8% of serotyped isolates from layer flocks. Additionally, SE constituted 50.0% of all *Salmonella* isolates serotyped from eggs [1].

It is widely acknowledged that the efficiency of *Salmonella* vaccination protocols in chickens can only be achieved when environmental pressure is low or non-existent [13]. However, cleaning and disinfection (C&D) procedures often fall short of satisfactory levels and fail to effectively eliminate infections. In caged layer houses, this challenge is partly attributed to the difficulty in cleaning cage tiers without the option of utilizing wet cleaning methods. Additionally, the persistent presence of rodent populations remains a common issue on many layer farms, perpetuating the infection cycle between flocks [14,15]. Numerous other studies have arrived at similar conclusions, highlighting persistent environmental contamination as the primary concern on commercial laying farms [15,16,17,18,19,20,21,22].

Chickens exposed to SE shortly after hatching can remain infected until they reach maturity, leading to the production of contaminated eggs and the potential spread of infection to susceptible, previously unexposed hens [23]. Consequently, it is crucial to maintain a *Salmonella*-free environment both inside and outside of poultry houses throughout the birds’ lifespan. A longitudinal study conducted in the UK involving 74 commercial layer flocks identified multiple serovars, with SE being the sole persistent serovar observed among single-age flocks. The study concluded that there is significant room for improvement in C&D procedures on many farms. Furthermore, the prevalence of *Salmonella*-positive samples obtained from wildlife vectors, including rodents, was 38.6%—more than double the prevalence found in samples from the houses themselves. This finding underscores the significant role played by wildlife vectors, particularly rodents, in transmitting *Salmonella* between successive flocks [10]. The carry-over of SE between successive laying flocks appears to be a common issue. Previous research has demonstrated the persistence of SE not only in empty houses but also in small pockets of litter and fan dust outside poultry facilities [24]. Despite advancements in hygiene measures and the availability of easier-to-clean housing facilities, reinfections and persistent *Salmonella* infections can still be observed in European layer farms.

In Germany, the housing of laying hens in colony cages is rare, and all cages must be phased out by the end of 2025 at the latest [25]. In 2017, the majority of laying hens in Germany, 58.1%, were kept in barn/floor systems, while 29.1% were in free-range systems. Only 5.6% of hens were housed in colony cages, and 23.0% were registered as organic [26]. Given this context, it was particularly interesting to investigate whether the carry-over of SE, which has been primarily described in relation to hens in conventional or colony cages, remained equally significant in the German system where only a small proportion (5.6%) of hens were housed in colony cages, and conventional cages were not in use.

Despite a declining trend in the prevalence of SE among German laying flocks in recent years, there were still 0.5% of flocks testing positive for SE in 2019 [27]. SE accounted for 45% of human salmonellosis cases in 2018 [28], underscoring its continued significance as both a prevalent serotype in laying hen flocks and a causative agent of human illness. EU legislation dictates that eggs from SE-positive hens cannot be sold as fresh table eggs but must undergo heat treatment. Consequently, an SE infection in a flock results in substantial financial losses for the producer, often necessitating the early depopulation of the flock and the implementation of rigorous and costly C&D procedures [29]. Moreover, recalls of affected poultry products further contribute to significant economic losses and damage to the producer’s reputation.

This study aimed to assess the potential occurrence of *Salmonella* carry-over between successive laying hen flocks on German farms and to evaluate the effectiveness of C&D procedures in floor-reared flocks. A total of ten farms were included in the study, and microbiological analyses were conducted on samples taken after the completion of C&D and before introducing new birds to the farms.

## 2. Materials and Methods

### 2.1. Farms

Ten commercial layer farms (32 houses) in north-west Germany (Lower Saxony and North Rhine Westphalia) that had previously reported SE-positive flocks were included in the current study. Farm sampling was conducted from July 2018 to March 2021, with one to three visits made to each farm. The farms varied in size, ranging from 20,000 to 160,000 birds, and utilized either the widely used commercial layer genetics from Hendrix (Hendrix Genetics BV, Boxmeer, The Netherlands) or Lohmann (Lohmann Breeders GmbH, Cuxhaven, Germany). The farms comprised one-to-four houses, and all the birds were floor-reared. Among the farms, five utilized a conventional floor system, four were free-range farms, and one was an organic farm.

### 2.2. Sampling Methods

Farms were visited during the downtime or service period, and specialized sampling methods were employed following C&D procedures to confirm the presence or absence of *Salmonella* species (spp).

During the farm visits, two different sampling methods were employed. First, Sterisox^®^ boot swabs (Sodibox^®^, Nevez, France) in size 46 pairs were utilized. These swabs were moistened with 10% tryptone salt broth (0.1% peptone water + 0.85% salt) to collect faecal samples from shoes covered with single-use plastic covers. Secondly, ready-to-use sampling towels (Sodibox^®^, Nevez, France) measuring 34 × 37 cm and moistened with 10% tryptone salt broth (0.1% peptone water + 0.85% salt) were used as handheld swabs to sample critical locations both inside and outside the poultry houses.

Both the boot swabs (4136/4137, SodiBox^®^, Nevez, France) and sampling towels (4030/4031, SodiBox^®^, Nevez, France) underwent ionization-based sterilization, with a minimum of 15 Kilogray. These sampling kits were manufactured in line with ISO11133 standards.

For each individual sample collection, a fresh pair of sterile nitrile gloves (Anhui Intco Medical Products Co., Ltd., Huaibei, China) was utilized to prevent potential cross-contamination among samples. The labelled samples were initially placed in a plastic bag and subsequently placed in an insulated transport container, which was cooled using frozen gel or ice packs. These containers were then promptly sent via express mail within 2–4 h after sampling to a specialized laboratory accredited under DIN EN ISO/IEC 17025 standards. The samples arrived at the laboratory within 24 h.

### 2.3. Sampling Techniques on Site

To gather samples from the floor, boot swabs were employed. Sampling within each house followed a specific pattern: starting from the bottom left corner of the house, the sampler walked across to the top right corner. Then, they proceeded along the top end to the top left corner and, from there, back to the bottom right corner, effectively crossing the house through the middle. This approach ensured that a representative area of the floor surface was covered. To guarantee a comprehensive sampling of the entire house, a minimum of 100 shuffling steps were taken.

Handheld swabs were collected following a thorough visual inspection of the facilities using a flashlight (Ledlenser P6R, Ledlenser GmbH & Co. KG, Solingen, Germany). Sampling was performed at critical points where visible residues of organic material were present, as well as other areas of interest known to have potential residual contamination based on field experience. Critical points typically refer to locations where proper C&D procedures may be challenging due to specific building characteristics, ongoing construction, inadequate building maintenance, or limited time between depopulation and restocking. Sampling points included water lines, feeders and feed pipes, floor cracks, nest boxes, carcass containers, waste belt systems, egg belts, electrical switchboards, and ventilation systems. During each visit, a range of 4 to 35 samples, consisting of boot swabs and towel samples, were collected.

### 2.4. Salmonella Isolation and Identification

The detection of *Salmonella* spp. was performed in accordance with DIN EN ISO 6579-1. For pre-enrichment, the swabs were immersed in pre-warmed buffered peptone water (BPW) (Merck, Darmstadt, Germany) and incubated at 37 ± 1 °C for 18 h. The swab samples were fully covered with peptone water during this process.

For cultivation, three droplets of the incubated BPW (≥0.1 mL in total) were pipetted separately onto a modified semi-solid Rappaport–Vassiliadis (MSRV) medium (Oxoid, Waltham, MA, USA). The plates were then incubated at 41.5 °C for 24 ± 3 h.

After the initial 24 ± 3 h of incubation, the plates were examined for any signs of growth. If no growth was observed, the plates were further incubated for an additional 24 ± 3 h and rechecked.

In the event that a swarming zone was detected, colonies were subcultured from the outer swarming zone onto agar modified with xylose lysine deoxycholate (XLD; Merck, Darmstadt, Germany) and Rambach agar (Merck, Darmstadt, Germany). The subcultures were then incubated at 34–38 °C for 24 ± 3 h. Presumptive *Salmonella* isolates were subsequently transferred onto Columbia agar (Merck, Darmstadt, Germany) with sheep blood (Thermo Scientific, Waltham, MA, USA) and incubated for 24 ± 3 h. The confirmation of *Salmonella* spp. was achieved using MALDI-TOF mass spectrometry (Bruker, Billerica, MA, USA) after this step.

### 2.5. Polymerase Chain Reaction

One millilitre of enriched peptone water was utilized for DNA extraction and PCR analysis using the Kylt^®^ *Salmonella* spp. DNA Extraction & Real-Time PCR Detection Kit, following the instructions outlined in the user’s manual (Kylt^®^ *Salmonella* spp. FLI-B 656, SAN Group Biotech Germany GmbH, Höltinghausen, Germany).

### 2.6. Serotyping

*Salmonella* serotyping was conducted using antisera (Sifin Diagnostics GmbH, Berlin, Germany) in accordance with the Kauffmann–White–Le Minor scheme, following the instructions provided in the user’s manual from Sifin GmbH [30].

The molecular profiles of four SE isolates were subjected to further analysis using pulsed field gel electrophoresis (PFGE). The protocol employed in the partner laboratory followed the standard operating procedure provided by the CDC in Atlanta, USA (PNL05, last updated December 2017). The complete protocol is available at https://www.cdc.gov/pulsenet/pathogens/pfge.html (accessed on 12 October 2018). For PFGE analysis, four strains originating from distinct regions but sharing the same slaughterhouse and transport resources were selected.

### 2.7. Statistical Analysis

The degree of flock contamination following C&D was evaluated by determining the average (weighted) percentage of positive samples. Chi-square tests were employed to evaluate differences in proportions and compare sample prevalence after disinfection. All statistical analyses were performed using JMP^®^ 16 (Statistical Discovery^TM^ from SAS, Marlow, SL7 2EB, UK).

Due to the limited number of farms in each housing system category (barn, free-range, and free-range organic), the free-range and free-range organic farms were combined into a single group for the purpose of statistical analysis.

## 3. Results

This study aimed to investigate the occurrence of SE in swabs collected from layer farms after the completion of the C&D procedure. A total of 244 samples were collected from ten-layer farms during the service period. The samples were tested for the presence of *Salmonella* using real-time *Salmonella* spp. PCR, followed by the microbiological culturing of any PCR-positive samples. At the time of sampling, all farms had a history of testing positive for SE during regular monitoring. The samples were taken from conventional floor-reared farms (n = 5), free-range farms (n = 4), and organically reared farms (n = 1). Sampling was conducted throughout the year, covering each season: spring (n = 53), summer (n = 78), fall (n = 89), and winter (n = 24).

### 3.1. Detection of Salmonella in Samples

**Figure 1:**
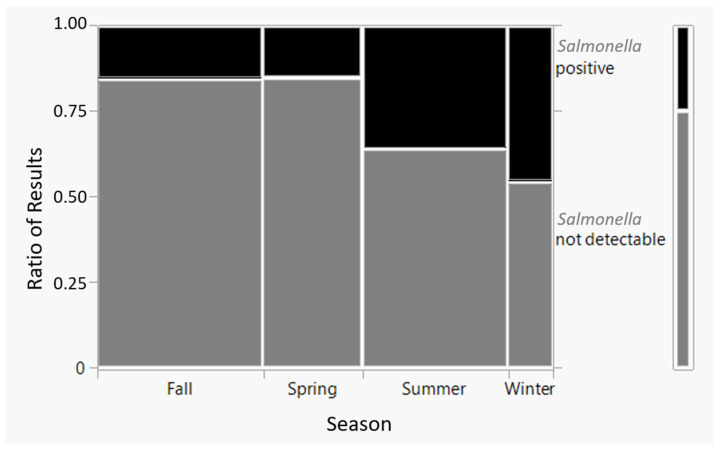
Chi-square test results on *Salmonella* prevalence vs. season.

A total of 25.0% (61 out of 244) of the collected samples tested positive using real-time PCR, and among those, 65.6% (40 out of 61) were confirmed by the culture (36 *Salmonella* Enteritidis and 4 rough *Salmonella* strains). This included 44 out of 185 (23.8%) samples from inside the poultry houses and 17 out of 59 (28.8%) samples from the surrounding areas outside the houses (Table 1). The prevalence of *Salmonella* was not significantly different between the inside and outside locations (chi-square, *p* = 0.4372). However, significant differences (*p* = 0.0006) in the occurrence of SE in collected samples were observed depending on the season (Figure 1). There were more *Salmonella*-positive samples in winter (11 out of 13; 84.6%) and summer (28 out of 50; 56%) compared to spring (eight out of 45; 17.8%) and autumn (14 out of 75; 18.7%).

During this study, *Salmonella* was detected in 30 out of 114 (26.3%) samples from floor-reared systems, 30 out of 112 (26.7%) from free-range systems, and 1 out of 18 (5.6%) from organic systems. The chi-square test indicated that there was no statistically significant difference (*p* = 0.1405) between barn-reared and free-range farms.

**Figure 2:**
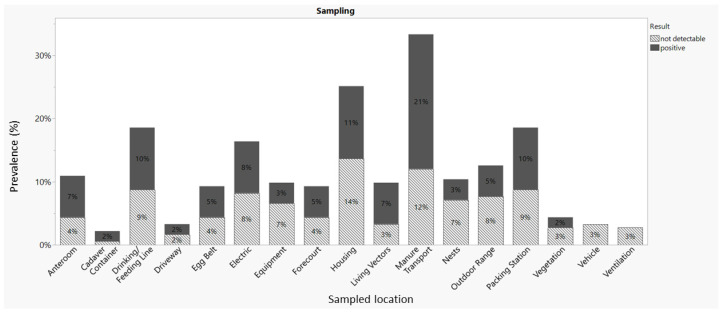
Comparison of SE prevalence rates based on sampled locations.

Samples were collected from various sources, including the anteroom, carcass container, boots, building structure, drinking/feeding line, driveway, egg belt, electrics, equipment, forecourt, living vectors, manure transport system, nests, outdoor range, packing station, and vegetation, and 48 out of 126 sample swabs tested positive for *Salmonella* DNA. However, no *Salmonella* DNA was detected in samples collected from vehicles (bicycle, car, and work vehicle) and the ventilation system (Table 2, Figure 2).

### 3.2. PFGE Typing of Salmonella Enteritidis

**Figure 3:**
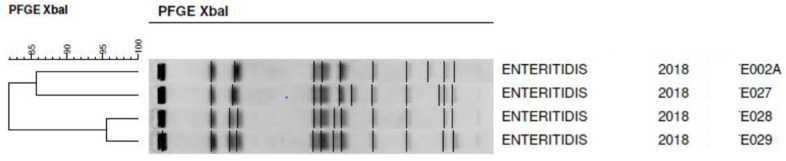
PFGE patterns of *Salmonella* Enteritidis isolates from four farms.

To rule out the possibility of the same SE strain being introduced into the farms through company-used crates, PFGE analysis was conducted to differentiate potentially different strains. All four strains selected for PFGE analysis exhibited distinct profiles (ENTE002A, ENTE027, ENTE028, and ENTE029) (Figure 3). Among these profiles, only ENTE002A was previously recorded in the laboratory database.

## 4. Discussion

Salmonellosis continues to be a significant foodborne pathogen, resulting in thousands of human illness cases annually throughout Europe and imposing a considerable economic burden on the entire poultry industry. The egg-producing sector, in particular, has experienced a substantial impact, despite the long-standing efforts of policymakers and the poultry industry to implement *Salmonella* surveillance and eradication programs for laying hens over the past decade.

Although EU-wide control measures for *Salmonella* in the layer industry have been successfully implemented since 2008, the number of human salmonellosis cases is no longer declining, and laying hen flocks infected with *S.* Enteritidis are still regularly discovered in various European countries. In Germany, for instance, 49 laying hen flocks (0.5% of flocks) were found to be infected with SE in 2019 [27]. Meanwhile, other European member states reported prevalence rates as high as 7.8% [1]. These figures are concerning, especially considering the mandatory control programs for SE and ST (*S.* Typhimurium) that have been in place across all EU member states since 2008. In Germany, all laying hens are required to be vaccinated against SE, and vaccination has undeniably been an effective measure in reducing the risk of *Salmonella* infection, leading to a significant decrease in both SE prevalence in chicken flocks and human cases [31].

Furthermore, continuous improvements in biosecurity and hygiene protocols have played a crucial role in controlling the spread of foodborne zoonoses. However, the transmission of *Salmonella* from one flock to another still occurs frequently and repeatedly. Most previous studies have focused on caged laying hen flocks, where C&D procedures are notoriously challenging. Therefore, it was of interest to investigate the extent of carry-over between consecutive flocks due to inadequate C&D in floor-reared flocks.

There are several reasons why the long-term elimination of *Salmonella* from laying hens may not be successful. In addition to factors like adaptability, virulence properties, persistence, and environmental factors (including transmission routes and reservoirs) significantly influence re-contamination with *Salmonella*. Previous studies have demonstrated that C&D procedures alone are often insufficient to prevent transmission or carry-over of *Salmonella* to new batches of birds [32]. Choosing an appropriate disinfection product and its proper application is crucial for ensuring effectiveness. However, the reluctance in some countries to use formaldehyde due to its carcinogenic properties has presented challenges in eliminating *Salmonella* since formaldehyde has been proven to be one of the most effective substances against it [33,34,35]. In this study, a variety of ready-to-use disinfectants (e.g., based on glutaraldehyde or peracetic acid) were applied. A comparison was not feasible as manufacturers varied and not all single components were known.

In a farm environment, it is widely accepted that cleaning alone removes approximately 90% of bacteria, while disinfection eliminates a further 6–7% [36]. The presence of organic material increases the survival time of microorganisms in the environment, and the level of organic matter on surfaces can impact the efficacy of chemical disinfectants [37]. A previous study has highlighted the importance of thorough cleaning with detergent prior to disinfection in reducing viral contamination on farms [38]. Therefore, the comprehensive removal of all organic matter is an essential step in the C&D process, and its significance should be emphasized to responsible individuals.

Our study clearly demonstrated that the abundance of positive samples detected within the poultry houses posed a substantial risk of infection for subsequent flocks. Furthermore, the significant percentage of positive samples in the surrounding environment also signifies a noteworthy risk. It is crucial to communicate to producers that, regardless of the initial level of biosecurity upon entering the houses, contamination can occur at any time, potentially leading to the infection of the flock.

We identified specific areas that pose challenges for cleaning, where dust tends to accumulate throughout the flock’s lifespan. Notably, electrical sockets were frequently found to be positive for *Salmonella*. Previous research has implicated dust as a potential source of *Salmonella*, contributing to flock-to-flock contamination [39]. In Japan, *Salmonella* was detected in nearly one-third of airborne dust samples from layer farms [40]. Therefore, cleaning these dusty areas becomes a critical vulnerability that must be addressed when sanitizing *Salmonella*-positive farms.

Areas and equipment exposed to faeces and dust appear to present a higher risk of *Salmonella* re-contamination. Interestingly, in our study, samples collected from the ventilation system tested negative for *Salmonella*. This finding was somewhat unexpected, considering that the ventilation system in laying houses is more complex compared to broiler houses. Collecting meaningful samples in a laying house without specialized equipment is challenging. Therefore, people collecting samples do not only need to be trained to understand potential sources of transmission, but also need testing equipment more adjusted to layer houses (e.g., telescope stick). By providing professional sample procedures including knowledge and equipment, false-negative samples might be avoided and carry-over could be prevented even more effectively.

*Salmonella* can also be transported outside through swirling dust. Depending on manure dust’s moisture content and particle size, viable *Salmonella* can still be detected for up to 291 days [41]. Consequently, the immediate surroundings of poultry houses, including roads and paths on the premises, can serve as persistent sources of reinfection through the presence of dust and faeces. The only way to prevent such reinfection is by strictly adhering to robust biosecurity measures when entering the poultry houses.

Previous studies have demonstrated that *Salmonella* can persist after C&D, with the highest likelihood of recovery from the floor, dropping boards/belts (in cage houses), and scratching areas (in non-cage houses) [42]. Our study detected substantial numbers of *Salmonella* in the manure transport system, the anteroom, and the drinking/feeding line. The carcass container and boots exhibited the highest prevalence at 50.0%, although only two samples from each location were included in this study. Additionally, many positive swabs were obtained from living vectors such as mice and wild birds. Notably, 43.0% of samples collected from living vectors tested positive for *Salmonella* during this study.

A previous study conducted on turkey farms showed a strong correlation between the percentage of positive samples after C&D and the likelihood of carry-over into the next flock. The specific location of positive samples did not appear to be as significant. However, having positive samples from the drinkers and feeders resulted in carry-over rates of 75% and 81.8%, respectively [32]. In our study, where one-third of the samples collected from the feeding and drinking lines tested positive, it is reasonable to assume a high probability of infection carry-over. The substantial percentage of positive swab samples in our study indicates the presence of a significant amount of residual *Salmonella* contamination on the premises just before the introduction of new birds. This contamination posed a considerable risk of overwhelming the birds’ protection conferred by vaccination. Unfortunately, assessing the *Salmonella* status of the follow-on flocks was not feasible, which could have provided valuable additional insights for the current study.

Furthermore, our study’s high percentage of positive samples from living vectors suggests that these vectors likely contributed to the carry-over of infection into the subsequent flock. However, even without considering the role of rodents, the number of positive swab samples after C&D alone would likely have been sufficient to result in carry-over.

In Germany, laying hen flocks are routinely vaccinated against SE. Nevertheless, it is widely acknowledged that vaccination alone is insufficient as the sole measure to combat infection with zoonotic *Salmonella* serovars. It is crucial to implement strict biosecurity measures in addition to vaccination in order to significantly reduce the risk of *Salmonella* incursion [8,43,44].

One limitation of this study is the lack of standardization in sampling, which was influenced by individual farm situations. However, implementing a strictly standardized sampling regime would primarily enhance the statistical power without fundamentally altering the results. Sampling was conducted on a farm-specific basis, primarily targeting critical control points that appeared to be inadequately cleaned upon visual inspection. Similar studies often face limitations regarding the number of samples that can be collected due to logistical and financial constraints. Since each farm is unique, sampling locations must be adapted based on the farm layout and the perceived importance of specific critical control points.

Considering the extensive experience of the sampler, the results effectively identify locations on the farms that should be inspected and considered as potential hotspots for *Salmonella* survival between flocks. While *Salmonella* can enter a farm through various pathways, the high percentage of positive samples following our study’s completion of the C&D procedure suggests that carry-over, rather than new introductions, seemed to be the primary issue. However, we wanted to rule out the possibility that transport boxes (plastic egg trays) used for safe egg transport might have been a common source of infection, distinct from carry-over. Consequently, four isolates from different regions but processed with the same loading and transport dispatcher were subjected to further investigation using PFGE. All isolates showed distinct genetic profiles according to PFGE, indicating different sources of origin. These findings support the assumption that the persistent presence of *Salmonella* in the environment and within the houses constituted the primary challenge that needed to be addressed.

In this study, in order to streamline the process and reduce the number of cultures, PCR was initially performed, and only PCR-positive samples were subsequently cultured to confirm the presence of viable *Salmonella* organisms and enable the typing of isolates. It is important to note that if the culture had been conducted on all swabs, we might have identified even more positive samples, as culturing is generally considered more sensitive than PCR (personal communication, Rob Davies, APHA Weybridge, UK). However, while performing culture experiments on all swabs could have resulted in a higher percentage of positive samples, the overall findings and conclusions would likely remain similar but with increased statistical significance.

The nearly identical proportion (approximately 26%) of positive samples detected both indoors and outdoors in our study underscores the significance of infection spread within the farm premises and the survival of *Salmonella* organisms in outdoor environments under various moisture conditions. While sanitation primarily focuses on C&D inside the poultry houses, the effective management of outdoor areas is equally critical and must be considered when aiming to eradicate *Salmonella* from a farm. Many studies primarily concentrate their sampling efforts on the interior of the houses, potentially overlooking the pockets of contamination in the immediate vicinity. Throughout our study, the regular sampling of swabs taken from outdoor areas, including manure transport tools, the forecourt, and living vectors, consistently revealed residual *Salmonella* contamination. Manure transport tools located outside represent significant potential for pathogen dissemination on the farm since they are frequently moved between houses and often bypass comprehensive C&D procedures. Vectors such as wild birds can contaminate the outdoor areas with infected faeces, while rodents can move between the inside of the houses and the outside, often eluding detection.

In this study, we observed a notable discrepancy in the percentage of *Salmonella*-positive samples depending on the season, although it remains uncertain whether this difference is an artifact or a genuine variation. It is known that foodborne bacteria are affected by climate change [45]. However, two previous studies found no evidence of seasonality in the occurrence of *Salmonella* infection in poultry [46,47]. While human salmonellosis cases typically peak during hot summer months due to changes in eating and cooking practices, as well as the increased multiplication of *Salmonella* on food due to higher temperatures, the prevalence of *Salmonella*-positive flocks does not appear to follow the same seasonal trend (personal communication, Rob Davies, APHA Weybridge, UK). In fact, certain factors might actually contribute to a higher prevalence during autumn months, such as rodent ingress on farms and increased condensation in feed mills, creating conditions that favour the survival and multiplication of *Salmonella* in feed. Accordingly, one study has demonstrated that SE reappeared in soil samples during cold winter weather [48]. Other studies have also shown that certain bacteria occur more seasonally in livestock facilities [49]. Since the identification of an infected flock is often not immediately linked to the initial occurrence of infection on the premises, there may be delays in detecting positive flocks, making it challenging to determine if *Salmonella* in poultry flocks exhibits a seasonal pattern or not.

If only a few samples are taken from easily cleanable and disinfectable areas, such as intact floor surfaces, there is a higher risk of overlooking residual contamination. As demonstrated in this study, achieving high sensitivity in detecting *Salmonella* requires an adequate number of samples and diligence in sampling techniques. It is crucial to employ comprehensive sampling techniques to ensure accurate and reliable results.

## 5. Conclusions

This study underscores the importance of the houses’ immediate surroundings for the carry-over of *Salmonella* between successive batches and the meticulous implementation of C&D procedures to eliminate residual bacteria from the interior of the houses. Despite the significant advancements made by the poultry industry in combating *Salmonella* over the past two decades, it is now more crucial than ever to educate farmers about these critical issues. Complacency regarding rushed rodent control C&D protocols and insufficient attention given to the environment surrounding the houses may be among the primary reasons why *S.* Enteritidis continues to be regularly detected in German laying hen flocks.

## Figures and Tables

**Table 1:** The prevalence of *Salmonella* (SE) for all samples taken from inside and outside of the farms (A–J).

Poultry Unit Code	Total No. of Samples	Inside		Outside	
		No. of Samples	No. Positive for *Salmonella* (%)	No. of Samples	No. Positive for *Salmonella* (%)
A (1)	39	30	5 (16.6%)	9	2 (22.2%)
B (2)	42	25	11 (44.0%)	17	7 (41.2%)
C (3)	19	17	5 (29.4%)	2	1 (50.0%)
D (4)	18	11	1 (9.1%)	7	0 (0.0%)
E (5)	18	12	1 (8.3%)	6	1 (16.7%)
F (6)	20	17	1 (5.9%)	3	2 (66.7%)
G (7)	13	9	2 (22.2%)	4	1 (25.0%)
H (8)	12	11	3 (27.3%)	1	0 (0.0%)
I (9)	4	1	0 (0.0%)	3	1 (33.3%)
J (10)	59	52	15 (28.8%)	7	2 (28.6%)
TOTAL	244	185	44 (23.8%)	59	17 (28.8%)

**Table 2:** The prevalence of *Salmonella* (SE) based on sampled location.

Type of Sample Site	No. of Samples	No. Positive for *Salmonella* (%)
Anteroom	12	4 (33.3%)
Boots	2	1 (50.0%)
Building structure (floor, walls etc.)	32	7 (21.9%)
Carcass container	2	1 (50.0%)
Drinking/feeding line	22	6 (27.3%)
Driveway	4	1 (25.0%)
Egg belt	11	3 (27.3%)
Electric	20	5 (25.0%)
Equipment	14	2 (14.3%)
Forecourt	11	3 (27.3%)
Living vectors (rodents, wild birds etc.)	7	3 (42.9%)
Manure transport	35	13 (37.1%)
Nests	15	2 (13.3%)
Outdoor range	17	3 (17.6%)
Packing station	22	6 (27.3%)
Vegetation	6	1 (16.7%)
Vehicles	6	0 (0.0%)
Ventilation	6	0 (0.0%)

## Data Availability

The data presented in this study are available on request from the corresponding author. The data are not publicly available due to data privacy reasons on farms.
